# Cold air exposure at − 15 °C induces more airway symptoms and epithelial stress during heavy exercise than rest without aggravated airway constriction

**DOI:** 10.1007/s00421-022-05004-3

**Published:** 2022-09-02

**Authors:** Linda M. Eklund, Åsa Sköndal, Ellen Tufvesson, Rita Sjöström, Lars Söderström, Helen G. Hanstock, Thomas Sandström, Nikolai Stenfors

**Affiliations:** 1 Division of Medicine, Department of Public Health and Clinical Medicine, Umeå University, Umeå, Sweden; 2 Department of Clinical Sciences Lund, Respiratory Medicine and Allergology, Lund University, Lund, Sweden; 3 Unit of Research, Education and Development, Department of Community Medicine and Rehabilitation, Umeå University, Östersund, Sweden; 4 Unit of Research, Education and Development, Östersund Hospital, Östersund, Sweden; 5 Department of Health Sciences, Swedish Winter Sports Research Centre, Mid Sweden University, Östersund, Sweden; 6 Department of Anesthesiology and Intensive Care, Östersund Hospital, Box 654, 831 27 Östersund, Sweden

**Keywords:** Cold temperature, Environmental chamber, Physical activity, Healthy, Respiratory physiology, Respiratory symptoms

## Abstract

**Purpose:**

Exposure to cold air may harm the airways. It is unclear to what extent heavy exercise adds to the cold-induced effects on peripheral airways, airway epithelium, and systemic immunity among healthy individuals. We investigated acute effects of heavy exercise in sub-zero temperatures on the healthy airways.

**Methods:**

Twenty-nine healthy individuals underwent whole body exposures to cold air in an environmental chamber at − 15 °C for 50 min on two occasions; a 35-min exercise protocol consisting of a 5-min warm-up followed by 2 × 15 min of running at 85% of *V*O_2_max vs. 50 min at rest. Lung function was measured by impulse oscillometry (IOS) and spirometry before and immediately after exposures. CC16 in plasma and urine, and cytokines in plasma were measured before and 60 min after exposures. Symptoms were surveyed pre-, during and post-trials.

**Results:**

FEV_1_ decreased after rest (− 0.10 ± 0.03 L, *p* < 0.001) and after exercise (− 0.06 ± 0.02 L, *p* = 0.012), with no difference between trials. Exercise in − 15 °C induced greater increases in lung reactance (X5; *p* = 0.023), plasma CC16 (*p* < 0.001) as well as plasma IL-8 (*p* < 0.001), compared to rest. Exercise induced more intense symptoms from the lower airways, whereas rest gave rise to more general symptoms.

**Conclusion:**

Heavy exercise during cold air exposure at − 15 °C induced signs of an airway constriction to a similar extent as rest in the same environment. However, biochemical signs of airway epithelial stress, cytokine responses, and symptoms from the lower airways were more pronounced after the exercise trial.

**Supplementary Information:**

The online version contains supplementary material available at 10.1007/s00421-022-05004-3.

## Introduction

Exposure to a cold climate has been shown to evoke numerous symptoms in humans (Nayha et al. 2011), as well as lead to an increased morbidity and mortality in the population (Group 1997; Rocklov and Forsberg 2008). Many different groups are affected, including children (Rasi et al. 2017), winter endurance athletes (Carlsen et al. 2008), while especially vulnerable are people with cardiopulmonary disease (Analitis et al. 2008) and the elderly (Schwartz 2005). The airways are sensitive to cold air exposure, which can trigger respiratory symptoms, bronchial hyperresponsiveness, bronchoconstriction, and asthma (Nayha et al. 2011; Rasi et al. 2017; Carlsen et al. 2008). To investigate the physiological effects of cold air in the airways of healthy individuals in a controlled environment with experimental exposure to sub-zero air gives us an opportunity to better understand the potentially harmful effects of a cold climate in vulnerable populations seen in previous studies.

Inhalation of cold air can cause airway inflammation, bronchial hyperresponsiveness (BHR), and bronchoconstriction (Carlsen et al. 2008). Subclinical bronchoconstriction (< 10% decrease in FEV_1_) can be triggered by cooling of the facial skin at rest (Koskela 2007). When high rates of minute ventilation are attained in winter endurance sports, the conditioning function of the nose is partly bypassed, and greater volumes of cold air reaches the lower airways, where it can trigger respiratory symptoms and BHR (Koskela 2007).

Experimental exposure to sub-zero temperatures can trigger bronchial obstruction in asthmatic individuals (Koskela et al. 1994; Koskela and Tukiainen 1995), but also in healthy people (Therminarias et al. 1998; Kennedy and Faulhaber 2018). In winter endurance athletes, exercise-induced bronchoconstriction (EIB) with > 10% decrease in FEV_1_ is commonly seen after hard exercise in sub-zero temperatures (Wilber et al. 2000; Kennedy et al. 2019). Thus, both asthmatic and non-asthmatic individuals are affected by the potentially harmful effects of the inhalation of sub-zero air. A more thorough assessment of the responses in healthy airways is a prerequisite for understanding the underlying mechanisms of respiratory disease onset as well as the cold air induced morbidity observed in subjects with pulmonary disease.

Club cell protein 16 (CC16) is expressed in different regions of the respiratory tract and is the main secretory protein of club cells located in the bronchioles (Hermans and Bernard 1999). Apart from the airways, CC16 has also been found in the human endometrium, kidneys and the prostate (Hermans and Bernard 1999). CC16 is proposed to function as a protective mediator against oxidative stress and inflammation in the airways (Broeckaert and Bernard 2000; Lakind et al. 2007; Gomes et al. 2011). Disruption of airway epithelial integrity increases the permeability of the epithelial barrier and leads to leakage of CC16 into blood and, through glomerular filtration, into urine (Broeckaert and Bernard 2000). Thus, the CC16 concentrations detected in blood originates almost exclusively from the respiratory tract and CC16 can therefore be regarded as a non-invasive marker of airway epithelial damage. CC16 levels after exposure to sub-zero temperatures have been sparsely investigated. Recently, we found that moderate exercise in − 10 °C, as well as in + 10 °C, increased plasma concentrations of CC16, with no significant difference between environments (Eklund et al. 2021).

Systemic immune effects of exercise in sub-zero temperatures are relatively unexplored with conflicting results regarding the potential impact on immune function and susceptibility to infections (LaVoy et al. 2011). Hard endurance exercise does increase numerous cytokines, such as interleukin (IL)-6, IL-8, and IL-10, thought to mainly function as immune regulators (Peake et al. 2015). How these immune responses are affected by sub-zero temperatures alone or in combination with exercise remains to be determined.

A wide range of symptoms have been described after moderate exercise and rest in a sub-zero environment in healthy individuals, but symptoms from the lower airways are typically reported predominantly by individuals with respiratory disease (Sjostrom et al. 2019). However, healthy female cross-country skiers can also experience cough following bouts of high-intensity exercise in sub-zero temperatures (Kennedy and Faulhaber 2018), as well as after a winter season of training and competing (Kennedy et al. 2016). Whether airway symptoms are associated with, or may predict, airway injury requires further investigation.

Although exercise in sub-zero temperatures is potentially harmful to the airways, it is unclear to what extent heavy exercise affects the peripheral airway, airway epithelium, and systemic immune responses among healthy individuals exposed to sub-zero temperatures. If we can better understand the cold-induced effects on the healthy respiratory tract with and without exercise, we will improve our understanding of the pathogenesis of cold-related morbidity and mortality seen in the population. Additionally, we lack evidence-based guidelines for effective threshold temperatures where the risk of adverse effects on the respiratory tract is minimized.

Our study aim was to investigate the acute effects of heavy exercise during whole-body exposure to sub-zero temperatures on the healthy airways, and to compare these with the airway effects of rest in the same environment. Our primary hypothesis was that exercise in − 15 °C would induce a greater reduction in forced expiratory volume in the first second (FEV_1_), compared to rest. Exploratory hypotheses were to investigate whether exercise in − 15 °C would induce more pronounced biochemical signs of epithelial stress, systemic inflammatory responses, and respiratory symptoms, compared to rest.

## Methods

### Study design

This was an experimental exposure study whereby study subjects rested and performed heavy exercise in sub-zero temperatures. In a crossover design, study subjects were exposed to − 15 °C for 50 min (min) in an environmental chamber on two occasions, while either exercising or resting. The order of the trials was randomized and the two exposures took place at least one week apart. The environmental chamber is located at the Swedish Winter Sports Research Centre, Mid Sweden University, Östersund, and has been described previously (Sjostrom et al. 2019). All the data were collected during May 2018, and the mean (range) outdoor temperature during this time was 11.4 (2.0–24.0) °C (SMHI 2018).

### Participants

Participants were recruited through local advertising. Inclusion criteria were: (1) age 18–65, (2) healthy without a medical history of allergy or asthma, (3) never smoker. Exclusion criteria were use of anti-inflammatory medication during the study period and lower airway infection < 4 weeks prior to the pre-test and exposures. A total of 29 individuals (11 female/18 male) completed the study. Four subjects used oral contraceptives. Participants were asked not to perform any strenuous exercise for 24 h before each test. Subjects were also requested to avoid caffeine and any exhausting means of transportation to the environmental chamber, on the day of each trial. Participants were instructed to dress warm, but layered clothing was recommended to be able to easily match the need of each exposure. Participant characteristics and lung function at baseline are presented in Table 1.

**Table 1:** Participant characteristics and baseline lung function

Characteristic	All *n* = 29	Female *n* = 11	Male *n* = 18
Age, year mean (range)	38 (19–62)	35 (19–62)	41 (26–50)
Height, cm	177 (10.2)	168 (6.0)	183 (7.2)
Weight, kg	74.4 (14.9)	63.0 (7.1)	81.3 (14.1)
Body mass index, kg/m^2^	23.5 (2.7)	22.4 (1.4)	24.1 (3.0)
VO_2_max, mL/kg/min	51.3 (8.2)	46.7 (5.0)	54.0 (8.6)
FEV_1_, % of predicted	102.9 (11.8)	100.7 (12.3)	104.3 (11.6)
FVC, % of predicted	101.4 (11.5)	99.1 (15.4)	102.8 (8.5)
R5Hz, % of predicted	101.6 (29.3)	103.3 (25.8)	100.5 (32.0)
R20Hz, % of predicted	116.3 (29.9)	123.4 (31.3)	112.0 (29.1)

Data presented as mean (SD) unless noted otherwise

*n* number, *VO*_*2*_*max* maximum rate of oxygen consumption, *FEV1* forced expiratory volume in 1 s, *FVC* forced vital capacity, *R5Hz and R20Hz* resistance at 5 and 20 Hz

### Pre-test

To determine maximum rate of oxygen consumption (*V*O_2_max), participants performed an endurance test prior to the exposures. The pre-test was designed as a submaximal and maximal ramp test, in which the participants progressively ran on a treadmill (Rodby Innovation, Vänge, Sweden) to volitional exhaustion. Oxygen consumption was recorded (AMIS 2001 model C, Innovision AS, Odense, Denmark), to determine the subject’s *V*O_2_max as well as *V*O_2_ at four pre-defined submaximal speeds. These measurements were used to calculate the speeds needed to elicit 60% and 85% of the participant’s *V*O_2_max at a 4% gradient during the exposures.

### Exposure protocol

**Fig. 1:**
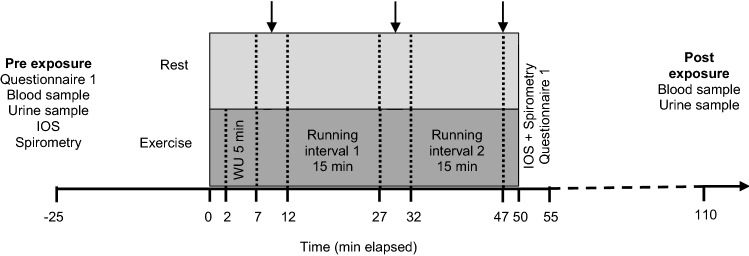
Exposure protocol. Study subjects were exposed to − 15 °C for 50 min (min) at two occasions in an environmental chamber, while either exercising or resting. Time 0 and 50 min indicates chamber entrance and exit, respectively. Arrows indicate collection of questionnaire 1 and 2. Chamber median (IQR) during the rest exposure: temperature − 15 (− 14.8 to − 15) °C; relative humidity 67.4 (63.9–72.9) %, absolute humidity 1.2 (1.2–1.4) g/m^3^. Chamber median (IQR) during the exercise exposure: temperature − 15 (− 14.8 to − 15) °C; relative humidity 69.9 (65.4–73.5) %, absolute humidity 1.3 (1.2–1.4) g/m^3^. The difference in relative humidity was significant between exposures (*p* < 0.001). *IOS* impulse oscillometry, *WU* warm-up

The subjects performed the two experimental exposures according to a pre-defined protocol described in Fig. 1. Each trial was 50 min. The exercise protocol consisted of warm-up (WU) at quick walking speed to elicit 60% of *V*O_2_max, as well as running interval 1 (R1) and 2 (R2) at 85% of *V*O_2_max. The running was performed on a motorized treadmill (Rodby Innovation, Vänge, Sweden) at a 4% gradient, with continuous monitoring of heart rate (model s610, Polar Electro Oy, Kempele, Finland). The rest protocol consisted of sedentary exposure.

Chamber data regarding humidity and temperature are presented in Fig. 1. Absolute humidity (*AbsH,* g/m^3^) was calculated based on the formula:

$$AbsH=\frac{6.112*rh*2.1674*e\frac{17.67*T}{T+273.15}}{T+273.15}$$, where *rh* (%) is relative humidity; and *T* (°C) is temperature (Sjostrom et al. 2019).

### Study variables

#### Lung function

Lung function was measured using (1) impulse oscillometry (IOS) (R5Hz and R20Hz, resistance at 5 and 20 Hz; X5Hz, reactance at 5 Hz; Z5Hz, respiratory impedance at 5 Hz; F_res_, resonance frequency Hz) which measures respiratory impedance, consisting of resistance and reactance, and can be used to isolate the peripheral lung resistance from the total airway resistance, and (2) spirometry (FEV_1_, forced expiratory volume in 1 s; FVC, forced vital capacity; FEV_1_/FVC, ratio of FEV_1_ to FVC), which measures flow and reflects resistance mainly in the central airways. During each trial, subjects performed IOS (Smith et al. 2005), followed by dynamic spirometry maneuvers (Jaeger Vyntus IOS, CareFusion, Germany), before entering the environmental chamber, as well as immediately after exiting the chamber. These timepoints were chosen since the maximal reduction in FEV_1_ after exercise usually occur within 5–10 min post-exercise (Hallstrand et al. 2018). Spirometric measurements followed the ATS/European Respiratory Society test criteria for acceptability and repeatability (Miller et al. 2005).

#### Biochemical markers

Before and 60 min after each exposure, blood and urine samples were collected. CC16 has been shown to increase in plasma up to 1 h after exercise (Tufvesson et al. 2013), hence our choice of time point. Blood was analyzed for differential cell counts and plasma was analyzed for CC16 and cytokines. Urine was analyzed for CC16 and corrected for creatinine. Of each male urine sample the first 100 ml was discarded to eliminate the effect of CC16 originating from the prostate (Andersson et al. 2007). Analyses of CC16 were performed with Human Clara Cell Protein ELISA kits (Biovendor, Modrice, Czech Republic). Cytokines in plasma were analyzed using high-sensitivity enzyme-linked immunosorbent assays (Human Quantikine ELISA kits, cat nr HS600C/HSTA00E, R&D systems, Minneapolis, MN, USA). Mean intra-assay coefficients of variation were < 5% for all plates.

#### Symptoms and subjective judgment of thermal stress

Symptoms and subjective judgment of the thermal conditions of the environmental chamber were assessed using two questionnaires and all variables inquired for are presented in Table 5 and supplemental Table 2. In the symptoms questionnaire (Sjostrom et al. 2019), symptom intensity was rated according to the Borg CR10-scale (Borg and Kaijser 2006) ranging from 0 (“no symptoms”) to 11 (“maximal symptoms”). Responses were collected at five time points: before exposure, after warm-up, after the first running interval, after the second running interval, and immediately after exiting the chamber (Fig. 1). Subjective judgment of the thermal conditions (ISO 10551:1995 questionnaire) (ISO 1995) was surveyed inside the environmental chamber at three time points: after warm-up, after the first running interval, and after the second running interval (Fig. 1). Differences in symptom intensity and subjective judgment of thermal stress between exposures were compared for all five/three time points (Table 5 and supplemental Table 2).

### Statistical analysis

A statistical power calculation was conducted using ΔFEV1 as the primary outcome variable. We assumed a mean (SD) of FEV1 = 4.58 (0.40) L, and that exercise in − 15 °C would decrease FEV1 by 6% compared to rest (Kennedy and Faulhaber 2018; Therminarias et al. 1998). Equal variance of FEV1 was assumed, and a correlation of 0.3 between exposures. With alpha at 0.05 and power at 0.80, 20 participants were required. Statistical analyses were performed using R (R. 2018).

The Shapiro–Wilk test and visual inspection was used to check for data distribution of continuous variables. For variables that were treated as normally distributed, a two-way repeated-measures ANOVA was performed to analyze the effects of time (pre/post) and trial (rest/exercise) on dependent variables. Variables with a skewed distribution were log-transformed before a two-way repeated-measures ANOVA was performed. When we had a main effect of time, we conducted a post hoc pairwise comparison using paired *t *tests and Wilcoxon signed-rank test, as we were interested in changes within trials. Furthermore, for lung function and CC16, we added sex as a between factor. For variables with a normal distribution, data are presented as mean (SD) and for variables with a non-normal distribution data are presented as median (interquartile range, IQR). A *p* value < 0.05 was considered statistically significant.

## Results

### Lung function

**Fig. 2:**
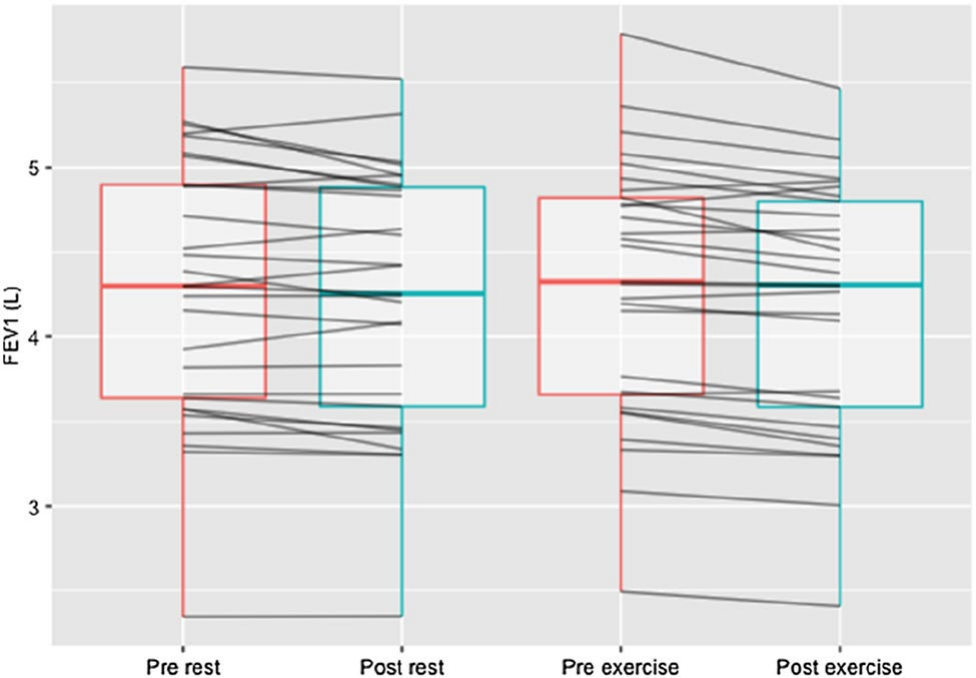
Individual changes in boxplot (mean, standard deviation) depicting a significant decrease in FEV_1_ pre- vs. post-rest (*p* < 0.001) and exercise (*p* = 0.012)

Lung function responses to rest and exercise are presented in Table 2 and individual changes in FEV_1_ in Fig. 2. There was a significant time x trial interaction for X5Hz (*F*(1, 28) = 8.90, *p* = 0.006, $\eta$^2^_g_ = 0.007), which was significantly altered following exercise (*p* = 0.023) but not rest (*p* = 0.529). There was a significant main effect of time on FEV1, FVC, and FEV1/FVC (FEV1: *F*(1, 28) = 29.31, *p* < 0.001, $\eta$^2^_g_ = 0.003; FVC: *F*(1, 28) = 6.97, *p* = 0.013, $\eta$^2^_g_ = 0.001; FEV1/FVC: *F*(1, 28) = 5.03, *p* = 0.033, $\eta$^2^_g_ = 0.003). Post-hoc pairwise comparisons, using paired *t*-tests, showed that FEV1 decreased during both rest (*p* < 0.001) and exercise (*p* = 0.012), FVC decreased only during rest (*p* < 0.001) but not during exercise (*p* = 0.469), and FEV1/FVC did not decrease during either rest (*p* = 0.064) or exercise (*p* = 0.152). There were no overall significant effects of exercise on lung function.

**Table 2:** Lung function responses to rest and exercise in − 15 °C

	Rest		Exercise		Time	Time × Trial
	Pre	Post	Pre	Post	*p* value	*p* value
FEV_1_ (L)	4.29 (0.77)	4.19 (0.74)*	4.30 (0.78)	4.24 (0.76)*	** < 0.001**	0.173
FVC (L)	5.43 (1.10)	5.34 (1.06)*	5.43 (1.11)	5.41 (1.08)	**0.013**	0.083
FEV_1_/FVC (%)	79.6 (5.91)	79.0 (5.61)	79.6 (5.77)	78.9 (6.19)	**0.033**	0.829
R 5 Hz (kPa/(L/s))	0.306 (0.089)	0.307 (0.080)	0.308 (0.075)	0.314 (0.069)	0.620	0.630
R 20 Hz (kPa/(L/s))	0.296 (0.078)	0.298 (0.078)	0.297 (0.073)	0.305 (0.067)	0.306	0.440
X 5 Hz (kPa/(L/s))	− 0.070 (0.024)	− 0.072 (0.024)	− 0.073 (0.024)	− 0.066 (0.022)*	0.233	**0.006**
Z 5 Hz (kPa/(L/s))	0.301 (0.082)	0.308 (0.080)	0.304 (0.073)	0.310 (0.070)	0.274	0.884
F_res_ (Hz)	10.286 (2.286)	10.578 (2.180)	10.617 (2.163)	10.684 (2.877)	0.598	0.680

Data presented as mean (SD). Significant *p* values in bold

*FEV*_*1*_ forced expiratory volume in 1 s, *FVC* forced vital capacity, *FEV*_*1*_*/FVC* ratio of FEV_1_ to FVC, *R5Hz and R20Hz* resistance at 5 and 20 Hz, *X5Hz* reactance at 5 Hz, *Z5Hz* respiratory impedance at 5 Hz, *F*_*res*_ resonance frequency Hz

*p < 0.05 vs. pre-trial measurement

For all lung function variables except for F_res_, there were significant main effects of sex (*p* < 0.030), as expected. There were no significant interactions between sex × time × trial for any of the lung function parameters.

### Club cell protein 16

**Fig. 3:**
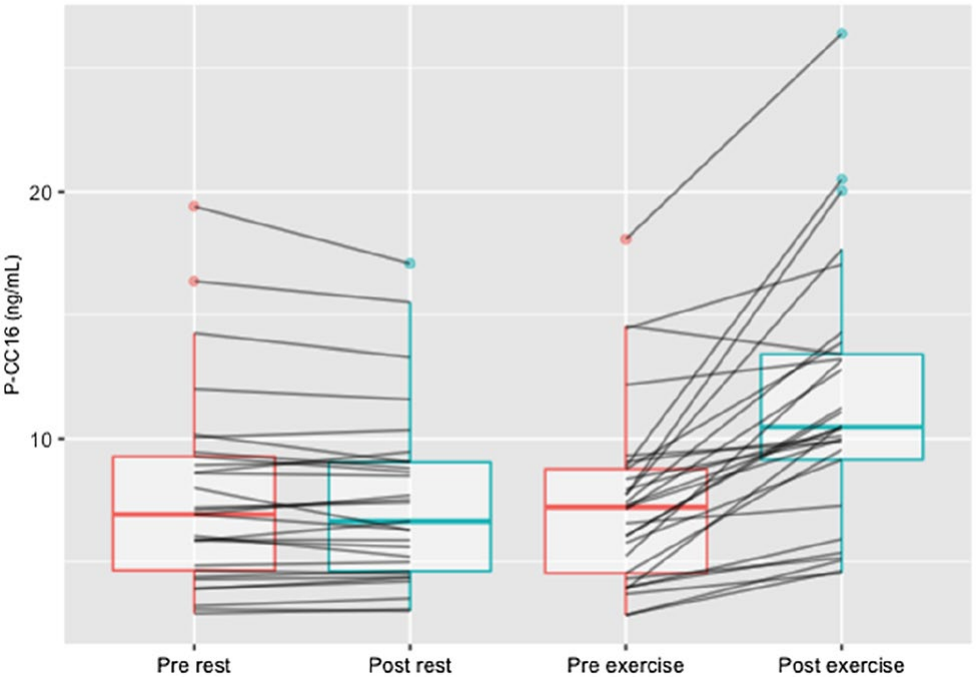
Individual changes in boxplot (median, interquartile range) depicting a significant increase in plasma CC16 pre- vs. post-exercise (*p* < 0.001), but not rest (*p* = 0.442)

There was a significant interaction between the effects of time and trial for CC16 in plasma (*F*(1, 28) = 40.84, *p* < 0.001, $\eta$^2^_g_ = 0.063), which was significantly altered following exercise (*p* < 0.001) but not rest (0.590). There was also a significant main effect of time on CC16 in plasma and urine (P-CC16: *F*(1, 28) = 52.78, *p* < 0.001, $\eta$^2^_g_ = 0.052; U-CC16: *F*(1, 28) = 5.74, *p* = 0.024, $\eta$^2^_g_ = 0.041). Post hoc pairwise comparisons, using Wilcoxon signed-rank test for paired data, showed that P-CC16 increased only during exercise (< 0.001) but not during rest (0.442). For U-CC16, the post hoc Wilcoxon signed-rank tests for paired data showed that it increased during both rest (*p* = 0.033) and exercise (*p* = 0.002). CC16 responses to rest and exercise in − 15 °C are presented in Table 3 and individual changes in P-CC16 in Fig. 3.

**Table 3:** Plasma and urine CC16 responses to rest and exercise in − 15 °C

	Rest		Exercise		Time	Trial	Time × Trial
	Pre	Post	Pre	Post	*p* value	*p* value	*p* value
P-CC16 (ng/mL)	6.9 (4.7–9.3)	6.6 (4.6–9.0)	7.2 (4.6–8.8)	10.5 (9.1–13.4)*	** < 0.001**	** < 0.001**	** < 0.001**
U-CC16/creatinine (ng/µmol creatinine)	0.5 (0.2–1.0)	0.6 (0.3–1.2)*	0.4 (0.2–1.0)	0.5 (0.3–2.0)*	**0.001**	0.132	0.104

Data presented as median (IQR). Significant *p* values in bold

*P-CC16* plasma CC16, *U-CC16* urinary CC16

*p < 0.05 vs. pre-trial measurement

There were no significant main effects or interactions of sex on plasma or urinary CC16.

### Cytokines

There was a significant time × trial interaction for IL-8 (*F*(1, 27) = 8.19, *p* = 0.008, $\eta$^2^_g_ = 0.020), which was significantly altered following exercise (*p* < 0.001) but not rest (*p* = 0.729). There was a significant main effect of time on IL-6, IL-8, and IL-10 (IL-6: *F*(1, 27) = 44.46, *p* < 0.001, $\eta$^2^_g_ = 0.077; IL-8: *F*(1, 27) = 36.26, *p* < 0.001, $\eta$^2^_g_ = 0.052; IL-10: *F*(1, 27) = 8.18, *p* = 0.008, $\eta$^2^_g_ = 0.016). Post-hoc pairwise comparisons, using Wilcoxon signed-rank test for paired data, showed that IL-6 increased significantly during both rest (*p* = 0.009) and exercise (*p* < 0.001), while IL-8 and IL-10 increased significantly only during exercise (IL-8 *p* < 0.001; IL-10 *p* = 0.003) but not during rest (IL-8 *p* = 0.728; IL-10 *p* = 0.779). A complete presentation of cytokine responses to rest and exercise are presented in Table 4.

**Table 4:** Plasma cytokine responses to rest and exercise in − 15 °C.

	Rest		Exercise		Time	Trial	Time × Trial
	Pre	Post	Pre	Post	*p* value	*p* value	*p* value
IL-1β (pg/ml)	0.4 (0.2–0.7)	0.5 (0.2–0.7)	0.3 (0.2–0.6)	0.3 (0.1–0.9)	0.401	0.082	0.396
IL-4 (pg/ml)	0.1 (0.1–0.2)	0.1 (0.1–0.2)	0.1 (0.1–0.2)	0.1 (0.1–0.1)	0.302	**0.043**	0.406
IL-5 (pg/ml)	1.1 (0.6–1.7)	1.0 (0.7–1.9)	0.9 (0.6–1.5)	0.8 (0.6–1.7)	0.856	0.206	0.385
IL-6 (pg/ml)	1.0 (0.6–1.4)	1.3 (0.8–2.0)*	0.8 (0.6–1.3)	1.3 (1.0–2.1)*	** < 0.001**	0.943	0.053
IL-8 (pg/ml)	3.1 (2.5–3.5)	2.9 (2.4–4.0)	2.9 (2.5–3.2)	4.0 (3.3–5.1)*	** < 0.001**	**0.019**	** < 0.001**
IL-10 (pg/ml)	0.5 (0.3–0.8)	0.6 (0.3–0.8)	0.5 (0.3–0.6)	0.6 (0.4–0.8)*	**0.008**	0.359	0.110
IL-13 (pg/ml)	5.8 (3.0–7.4)	6.5 (5.6–8.5)	3.5 (2.3–5.9)	5.3 (3.4–9.4)	0.222	**0.032**	0.862
IL-25 (17E) (pg/ml)	1.2 (0.8–1.8)	1.2 (0.5–1.7)	0.8 (0.5–1.3)	0.9 (0.5–1.4)	0.088	**0.037**	0.897
GM-CSF (pg/ml)	0.2 (0.1–0.3)	0.2 (0.1–0.4)	0.2 (0.1–0.3)	0.2 (0.1–0.3)	0.932	0.978	0.911
TNFα (pg/ml)	2.8 (2.1–3.8)	3.4 (1.9–4.2)	2.6 (2.0–3.3)	2.5 (1.9–3.7)	0.202	**0.014**	0.466

Data presented as median (IQR). Significant *p* values in bold

*IL-1β* interleukin 1 beta, *IL-4* interleukin 4, *IL-5* interleukin 5, *IL-6* interleukin 6, *IL-8* interleukin 8, *IL-10* interleukin 10, *IL-13* interleukin 13, *IL-25 (17E)* interleukin 25 (also known as interleukin 17E), *GM-CSF* granulocyte–macrophage colony-stimulating factor, *TNFα* tumor necrosis factor alfa

*p < 0.05 vs. pre-trial measurement

### Symptoms

Symptom intensity before entering the chamber did not differ between trials. Exercise in − 15 °C induced a significantly greater symptom score for rhinitis, irritation in the throat, irritation in the chest, feeling warm, and breathlessness, compared to the rest trial. Symptoms that were significantly more prominent in the rest trial were cold face, cold extremities, and physical discomfort. A comparison of all symptoms and intensity in rest vs. exercise is presented in Table 5.

**Table 5:** Comparison of symptom intensity using Borg CR10-scale (Borg and Kaijser 2006) between exposures, rest vs. exercise in − 15 °C.

	Rest	Exercise	*p* value^a^
Rhinitis			
Pre^b^	0.5 (0.0–1.0)	1.0 (0.5–1.0)	0.457
Warm-up^c^	1.0 (0.5–2.0)	2.0 (1.0–2.0)	**0.002**
Running interval 1^d^	2.0 (1.0–4.0)	3.0 (3.0–5.0)	** < 0.001**
Running interval 2^e^	3.0 (1.0–5.0)	4.0 (3.0–5.0)	**0.023**
Post^f^	2.0 (0.5–2.0)	1.0 (0.5–2.0)	0.084
Irritation in the nose			
Pre^b^	0.0 (0.0–0.5)	0.0 (0.0–0.5)	0.601
Warm-up^c^	1.0 (0.0–2.0)	1.0 (0.0–2.0)	0.611
Running interval 1^d^	2.0 (0.0–4.0)	1.0 (0.0–3.0)	0.744
Running interval 2^e^	3.0 (0.5–4.0)	1.0 (0.5–2.0)	**0.032**
Post^f^	0.5 (0.0–2.0)	0.0 (0.0–0.5)	0.078
Irritation in the throat			
Pre^b^	0.0 (0.0–0.5)	0.0 (0.0–1.0)	0.846
Warm-up^c^	0.0 (0.0–0.5)	0.0 (0.0–1.0)	**0.003**
Running interval 1^d^	0.0 (0.0–2.0)	2.0 (0.5–3.0)	** < 0.001**
Running interval 2^e^	0.0 (0.0–2.0)	1.0 (0.0–3.0)	**0.001**
Post^f^	0.0 (0.0–1.0)	0.0 (0.5–2.0)	**0.016**
Irritation in the chest			
Pre^b^	0.0 (0.0–0.0)	0.0 (0.0–0.0)	1.000
Warm-up^c^	0.0 (0.0–0.0)	0.0 (0.0–0.5)	0.065
Running interval 1^d^	0.0 (0.0–0.0)	0.0 (0.0–1.0)	**0.014**
Running interval 2^e^	0.0 (0.0–0.0)	0.0 (0.0–1.0)	**0.002**
Post^f^	0.0 (0.0–0.5)	0.0 (0.0–1.0)	**0.012**
Cold face			
Pre^b^	0.0 (0.0–0.0)	0.0 (0.0–0.0)	1.000
Warm-up^c^	1.0 (0.5–3.0)	2.0 (0.5–3.0)	0.502
Running interval 1^d^	3.0 (2.0–4.0)	1.0 (1.0–3.0)	** < 0.001**
Running interval 2^e^	3.0 (2.0–5.0)	1.0 (0.5–3.0)	** < 0.001**
Post^f^	0.0 (0.5–2.0)	0.0 (0.0–0.0)	**0.011**
Cold extremities			
Pre^b^	0.0 (0.0–0.0)	0.0 (0.0–0.0)	0.408
Warm-up^c^	1.0 (0.5–1.0)	1.0 (0.5–3.0)	**0.006**
Running interval 1^d^	3.0 (2.0–5.0)	1.0 (0.5–3.0)	**0.005**
Running interval 2^e^	5.0 (3.0–6.0)	1.0 (0.0–3.0)	** < 0.001**
Post^f^	2.0 (2.0–4.0)	0.0 (0.0–0.5)	** < 0.001**
Physical discomfort			
Pre^b^	0.0 (0.0–0.0)	0.0 (0.0–0.0)	1.000
Warm-up^c^	0.5 (0.0–1.0)	1.0 (0.0–2.0)	**0.019**
Running interval 1^d^	2.0 (1.0–4.0)	1.0 (0.0–2.0)	0.160
Running interval 2^e^	4.0 (3.0–5.0)	1.0 (0.0–3.0)	**0.002**
Post^f^	1.0 (0.0–2.0)	0.0 (0.0–0.5)	**0.010**
Feeling warm			
Pre^b^	1.0 (0.0–3.0)	2.0 (0.0–3.0)	0.984
Warm-up^c^	0.0 (0.0–2.0)	0.5 (0.0–1.0)	0.724
Running interval 1^d^	0.0 (0.0–1.0)	3.0 (2.0–4.0)	**0.001**
Running interval 2^e^	0.0 (0.0–2.0)	4.0 (3.0–5.0)	** < 0.001**
Post^f^	0.0 (0.0–2.0)	3.0 (1.0–5.0)	** < 0.001**
Breathlessness			
Pre^b^	0.0 (0.0–0.0)	0.0 (0.0–0.0)	0.386
Warm-up^c^	0.0 (0.0–0.0)	1.0 (0.5–2.0)	** < 0.001**
Running interval 1^d^	0.0 (0.0–0.0)	3.0 (2.0–4.0)	** < 0.001**
Running interval 2^e^	0.0 (0.0–0.0)	4.0 (2.0–4.0)	** < 0.001**
Post^f^	0.0 (0.0–0.0)	0.0 (0.0–0.5)	**0.004**

Data presented as median (IQR). Significant *p* values in bold

^a^Comparison of symptom intensity, rest vs. exercise

^b^Before entering the chamber

^c^After warm-up

^d^After first running interval

^e^After second running interval

^f^After exiting the chamber

### Other variables

Differential cell count responses to rest and exercise in − 15 °C are presented in supplemental Table 1.

A comparison and description of participant perception of thermal conditions in the environmental chamber is presented in supplemental Table 2.

## Discussion

This study investigated airway effects of heavy exercise vs. rest in − 15 °C. In this experimental study we mimicked endurance training in cold climates using whole body exposures in an environmental chamber and a 50-min protocol consisting of 35 min exercise with 5-min warm-up followed by 2 × 15 min of running at 85% of *V*O_2_max. Rest in − 15 °C induced signs of a proximal airway constriction and airway epithelial stress, it also caused an increase in pro-inflammatory cytokines and gave rise to general symptoms. Adding exercise did not induce an aggravated airway constriction compared to rest. However, exercise induced more pronounced biochemical signs of airway epithelial stress, a more extensive cytokine response, as well as symptoms from the lower airways, compared to rest.

Our study shows that whole body exposure to − 15 °C with heavy exercise as well as rest elicits signs of proximal airway constriction, measured by a decrease in FEV_1_, in healthy individuals. The reduction in FEV_1_ was < 10% and thus below the criterion for clinical bronchoconstriction. Interestingly, the rest exposure induced a decrease in FEV_1_ of a similar magnitude to exercise, as well as a significant decrease in FVC, indicating that temperature and humidity may play a role in inducing bronchoconstriction, independently of exercise hyperpnea. Although hydration level of study participants was not measured in the present study, cold air is believed to have a dehydrating effect on the airways (Anderson and Daviskas 2000).

Our findings are in line with previous studies and various mechanisms have been proposed to explain cold-associated effects on the airways. One hypothesis is that an increased ventilation with cold air can accelerate evaporation of the protective airway surface liquid lining the respiratory tract, leading to dehydration of the airways, a hyperosmolar milieu, and release of inflammatory mediators resulting in smooth muscle contraction and bronchoconstriction in susceptible individuals (Carlsen 2012; Anderson and Daviskas 2000). One of the mechanisms in response to the decreased levels of airway surface liquid is an increased capillary leakage of plasma. Another hypothesis suggests that repeated exposure of the airway smooth muscle to components leaked in plasma may alter its contractile properties, leading to the development of hyperresponsiveness (Anderson and Kippelen 2005). Sustained and repeated inhalation of large volumes of cold, dry air is believed to be the main cause of bronchial hyperresponsiveness and exercise-induced asthma in winter endurance athletes (Carlsen et al. 2008; Hanstock et al. 2020).

Clinical studies support the theory of an inflammatory response in the airways to exercise in cold air, in healthy as well as in vulnerable individuals. Elevated levels of inflammatory cells, such as granulocytes and macrophages, have been found in bronchoalveolar lavage fluid from the lower airways in healthy subjects after mild exercise in − 23 °C (Larsson et al. 1998). Also, airway inflammatory markers have been shown to increase in sputum after exposure of asthmatic individuals to − 5 °C (Seys et al. 2013), as well as in non-asthmatic female cross-country skiers during the winter season (Kennedy et al. 2016).

Regarding the theory of cold-induced hyperresponsive airway smooth muscle (Anderson and Kippelen 2005), the underlying mechanisms may be multifactorial. A slight bronchoconstriction, with a maximal fall in FEV_1_ of 5.8 ± 0.8% (mean ± standard error), can be provoked by cooling of the facial skin in sub-zero temperatures, in healthy individuals as well as in subjects with respiratory disease (Koskela and Tukiainen 1995), suggesting this to be a reflex mechanism to a trigger stimulus rather than a pathophysiological response. However, this effect is potentiated as exercise intensity increases. Bouts of heavy exercise in cold temperatures ranging from 0 to − 20 °C have been shown to decrease FEV_1_ up to ~ 25% in healthy, female athletes, with no significant differences between temperatures (Kennedy and Faulhaber 2018). However, in a study on healthy young men, no reduction of FEV_1_ was detected after short-duration moderate-intensity exercise in − 20 °C (Pekkarinen et al. 1989). Possible explanations for the divergence of these results include differences in exercise intensity and ventilation rates. A high minute ventilation is one of the two most important factors in triggering cold air-provoked respiratory symptoms, besides individual susceptibility (Koskela 2007).

By including impulse oscillometry, we found that heavy exercise in − 15 °C induced a significant increase in lung reactance at 5 Hz (X5Hz), indicating peripheral bronchodilatation, that was also significantly different between trials. Reactance (X) is a measurement of the elastic properties of the peripheral lung and the inertia of the air movement through the conducting airways. X5Hz uses a low frequency to detect changes in the peripheral lung. It has long been established that exercise has a bronchodilator effect through a reduction of vagal tone in combination with β-receptor stimulation by circulating catecholamines (Antonelli et al. 2012). However, based on previous studies (Koskela 2007), we hypothesized that heavy exercise in − 15 °C would induce signs of bronchoconstriction and that these would be more prominent than after exposure during rest, yet we found no differences between exposures. We believe this may be due to the increase in lung elastance counteracting the cold-induced proximal bronchoconstriction. Based on our findings from the use of both impulse oscillometry and spirometry, the airway effects of exercise in sub-zero temperatures appear more intricate than previously detected.

Whole body exposure to cold air at − 15 °C induced significant signs of airway epithelial stress measured by Club Cell protein 16 (CC16) in urine, after exercise as well as rest. Plasma CC16 significantly increased after exercise in − 15 °C, but not after rest. The fact that urinary CC16 significantly increased also after the rest trial is interesting. To our knowledge, this was the first study to investigate CC16 levels after rest in a sub-zero environment and our findings indicate that sub-zero temperature and low humidity can affect airway epithelial integrity, regardless of activity level.

CC16 is suggested to have anti-inflammatory/oxidative effects and work as a mediator in the pulmonary inflammatory response (Broeckaert and Bernard 2000). Elevated CC16 levels can be detected in serum as a result of an increased permeability following damage to the airway epithelial barrier (Broeckaert and Bernard 2000). CC16 has therefore gained ground as a biochemical marker for airway epithelial integrity and different factors have been shown to correlate to increased CC16 levels. Condition of the inspired air is one such factor, with increased levels after eucapnic hyperventilation (Bolger et al. 2011b), as well as after exposure to cold, dry air compared to warm, humid air (Bolger et al. 2011a). Moderate- to high-intensity exercise increases CC16 in healthy adults in serum/plasma and urine in warm environments (Chimenti et al. 2010; Tufvesson et al. 2013), in plasma in sub-zero temperatures (Eklund et al. 2021), as well as in nasal lavage in a hot, ozone-polluted environment (Gomes et al. 2011). A high minute ventilation leading to dehydration of the airways has been proposed to cause the elevated CC16 levels seen after exercise (Tufvesson et al. 2013). During rest and tidal breathing, less air reaches the distal airways containing the CC16-producing Club cells (Broeckaert and Bernard 2000). With the raised ventilatory demands during exercise, a larger proportion of the airways are recruited (Broeckaert and Bernard 2000), which might explain the increase in plasma CC16 levels seen in the present study after exercise, and the absence of this increase after rest. Previous studies have shown large differences in urinary CC16 levels after exercise (Bolger et al. 2011a; Tufvesson et al. 2013), suggesting this to be an expression of glomerular filtration differences and therefore not entirely a reflection of the degree of epithelial stress in the respiratory tract. Whether changes in CC16 levels then correspond to lung function effects has been sparsely investigated. A review of CC16 as a marker of pulmonary injury after exposure to a broad range of investigated irritants found no correlation between transient elevated CC16 levels and impaired lung function (Lakind et al. 2007). Our findings are in line with previous studies, showing that heavy exercise during whole body exposure to -15 °C leads to an increased airway epithelial stress compared to rest in the same environment, without signs of aggravated airway obstruction.

It has long been established that excessive inhalation of cold air can cause a local inflammatory response in the airways (Sue-Chu et al. 1998, 1999; Karjalainen et al. 2000), but the systemic immune effects of exercise in sub-zero temperatures are less well explored. IL-6, IL-8, and IL-10 increased after heavy exercise in − 15 °C, which was expected and is consistent with existing data (Peake et al. 2015). Increased gene expression of these cytokines in skeletal muscle may partly explain the elevated levels seen after endurance exercise, which are thought to correlate to exercise intensity and the extent of muscle damage (Peake et al. 2015). Therefore, it is interesting that the IL-6 rise after rest was of a similar magnitude to the response seen after exercise. IL-6 is thought to play a role in thermoregulation by contributing to maintenance of core temperature, as well as initiating a fever response during infection (Egecioglu et al. 2018). IL-6 knockout mice exposed to 4 °C for 6 days had a lower body temperature than wild-type controls, indicating thermogenesis was partly regulated by IL-6 (Egecioglu et al. 2018). Our findings indicate that the cold-induced elevation of IL-6 levels is more prominent than the exercise-induced upregulation in skeletal muscle. The involvement of IL-6 in upholding core body temperature during cold exposure in humans requires further investigation.

IL-8 can be released from airway epithelial and smooth muscle cells, as well as from skeletal muscle, as a response to stress, and has therefore been proposed as a biomarker of airway epithelial cell injury (Kippelen and Anderson 2012). In a study on healthy individuals completing a half-marathon, increased IL-8 concentrations were found in sputum (Chimenti et al. 2010). A recent study on healthy men performing 20 min of intense cycling in an environmental chamber showed increased salivary IL-8 concentrations after exercise in normal conditions (20 °C, 60% relative humidity), but not after exercise in sub-zero temperatures (− 20 °C, 40% relative humidity), although the difference between trials was non-significant (Patlan et al. 2021). As the authors speculate (Patlan et al. 2021), exercise intensity and duration are likely to play roles in the cold-induced upregulation of IL-8 in the airways. In our study, the increased levels of IL-8 observed in healthy individuals indicate a non-specific systemic inflammatory response to exercise in sub-zero temperatures, although the correlation with airway thermal stress remains to be explored.

Healthy individuals reported a broad range of symptoms after whole body exposure to − 15 °C in both trials. Exercise in − 15 °C induced symptoms specific to the lower airways, such as irritation in the chest and breathlessness, which were completely absent during rest in the environmental chamber. Symptoms that were more prominent during the rest trial were of a more general character, such as cold sensations and physical discomfort. Moderate exercise in sub-zero temperatures has not previously been shown to induce symptoms from the lower airways in healthy subjects (Sjostrom et al. 2019; Eklund et al. 2021), whereas high-intensity exercise has (Kennedy et al. 2016; Kennedy and Faulhaber 2018), supporting the theory that with increased ventilation demands, more incompletely conditioned air will reach the lower respiratory tract, thus cooling and dehydrating the airways (Koskela 2007). Compared to rest, heavy exercise in − 15 °C induced more symptoms from the lower airways, which corresponds well with our findings of epithelial stress and signs of a slight bronchoconstriction. The absence of symptoms from the lower respiratory tract during the rest trial is also reflected in the lower magnitude of changes in biomarker levels seen after this trial. This is also well in line with previous epidemiological research on cold exposure, where healthy individuals mainly reported musculoskeletal symptoms (Nayha et al. 2011).

### Strengths and limitations

This study used whole-body exposures in an environmental chamber with reliable and measurable conditions in an attempt to simulate outdoor exposure to cold climates. By the use of lung function measurements, biochemical markers, and symptoms we have taken a comprehensive approach in investigating effects of sub-zero temperatures on the healthy airways. Considering that multiple measurements increase the risk of type 1 error, the results concerning the non-primary outcomes are to be considered exploratory. The absence of sex differences within and between trials should be interpreted with caution, as the study was not powered to detect sex differences. With such low absolute humidity, small changes in environmental water vapor can substantially affect relative humidity. The small difference in relative humidity we found was likely an artifact of the chamber environment in that more humidity accumulated due to the increased minute ventilation during exercise compared to rest. The resulting water vapor could not be evacuated quickly enough from the chamber so as not to affect relative humidity.

## Conclusion

This study shows that whole-body exposure to cold air at − 15 °C during rest affects the airways, inducing low-grade bronchoconstriction and signs of epithelial stress, but did not give rise to symptoms from the lower respiratory tract in healthy subjects. Adding heavy exercise did not induce any signs of aggravated airway constriction compared to rest during cold exposure. However, heavy exercise did induce intricate effects on the airways in healthy subjects with more pronounced biochemical signs of airway epithelial stress, a more extensive cytokine response, as well as symptoms from the lower airways, compared to rest. Even though our results were mild and acute, it is possible that if the stress is regularly repeated the effects will be accumulated, enhanced, and lead to clinical manifestations such as asthma. Also, in individuals with pre-existing pulmonary disease these responses might have a larger clinical impact and so exposure to − 15 °C even at rest could be disadvantageous. Further studies regarding the effects of exercise in sub-zero temperatures in vulnerable subjects are warranted.

## Supplementary Information

Below is the link to the electronic supplementary material.

Supplementary file1 (DOCX 31 KB)

## Data Availability

The datasets generated during the current study are available from the corresponding author on reasonable request.

## Abbreviations

Abs H: Absolute humidity
ANOVA: Analysis of variance
BHR: Bronchial hyperresponsiveness
CC16: Club cell protein 16
CI: Confidence interval
EIB: Exercise-induced bronchoconstriction
FEV_1_: Forced expiratory volume in one second
F_res_: Resonance frequency
FVC: Forced vital capacity
GM-CSF: Granulocyte–macrophage colony-stimulating factor
HCT: Hematocrit
IL: Interleukin
IOS: Impulse oscillometry
IQR: Interquartile range
MCH: Mean cell hemoglobin
MCHC: Mean cell hemoglobin concentration
MCV: Mean cell volume
*n*: Number
R: Resistance
RBC: Red blood cell count
R1: Running interval 1
R2: Running interval 2
rh: Relative humidity
SD: Standard deviation
T: Temperature
TNFα: Tumor necrosis factor alfa
*V*O_2_max: Maximum rate of oxygen consumption
WU: Warm-up
X: Reactance
Z: Respiratory impedance
