# Supplementary material for: Cold air exposure at − 15 °C induces more airway symptoms and epithelial stress during heavy exercise than rest without aggravated airway constriction
Source: Eur J Appl Physiol. 2022 Sep 2;122(12):2533–44. doi: 10.1007/s00421-022-05004-3 (PMC9613713; doi:10.1007/s00421-022-05004-3)
Supplement: Supplementary file 1 — Supplementary file1 (DOCX 31 KB) [file 421_2022_5004_MOESM1_ESM.docx]

**Title** Cold air exposure at -15 °C induces more airway symptoms and epithelial stress during heavy exercise than rest without aggravated airway constriction

**Author list**Linda M. Eklund MD^1^ ORCID: 0000-0003-3739-0084, Åsa Sköndal MD, Ellen Tufvesson Assoc Prof^2^ ORCID: **0000-0002-4941-332X**, Rita Sjöström RPT, Ass Prof^3^ ORCID: 0000-0003-3466-5150, Lars Söderström retd Sr Stat^4^ ORCID: 0000-0002-6474-6501, Helen G. Hanstock PhD^5^ ORCID: 0000-0002-5381-736X, Thomas Sandström MD, Prof^1^ ORCID 0000-0002-0174-0882, Nikolai Stenfors MD, Assoc Prof^1^ ORCID: 0000-0002-1684-1301

**Affiliations**

^1^Division of Medicine, Department of Public Health and Clinical Medicine, Umeå University, Umeå, Sweden

^2^Department of Clinical Sciences Lund, Respiratory Medicine and Allergology, Lund University, Lund, Sweden

^3^Department of Community Medicine and Rehabilitation, Unit of Research, Education and Development, Östersund, Umeå University, Sweden

^4^Unit of Research, Education and Development, Östersund Hospital, Östersund, Sweden

^5^Swedish Winter Sports Research Centre, Department of Health Sciences, Mid Sweden University, Östersund, Sweden

**Corresponding author** Linda Eklund, Department of Anesthesiology and Intensive Care, Box 654, Östersund Hospital, 831 27 Östersund, Sweden. Email: [linda.eklund@umu.se](mailto:linda.eklund@umu.se)

## Supplementary Material

**Table 1** Blood cell count responses to rest and exercise in -15 °C. Data presented as median (IQR). Significant p-values in bold.

|  | Rest | | Exercise | | Time | Trial | Time x Trial |
| --- | --- | --- | --- | --- | --- | --- | --- |
|  | Pre | Post | Pre | Post | P-value | P-value | P-value |
| Hemoglobin  (g/L) | 136.5  (133.0-142.2) | 138.5 (132.8-141.2) | 139.5 (132.5-144.2) | 137.5  (133.0-  141.5) | 0.465 | 0.430 | 0.111 |
| RBC  (x 10^9^/L) | 4.52  (4.42-4.78) | 4.59  (4.44-4.72) | 4.69  (4.49-4.81) | 4.62  (4.47-4.78) | 0.315 | 0.184 | 0.351 |
| HCT | 0.41  (0.40- 0.43) | 0.42  (0.40- 0.43) | 0.43  (0.40- 0.44) | 0.42  (0.40-0.42) | 0.099 | 0.319 | 0.118 |
| MCV  (fL) | 91.0  (88.8-92.0) | 91.0  (88.8-92.3) | 91.0  (89.0-92.0) | 90.0  (88.0-92.0) | 0.131 | 0.223 | 0.166 |
| MCH  (pg) | 30.0  (29.0-30.3) | 30.0  (29.0-30.3) | 30.0  (29.0-30.3) | 30.0  (29.0-30.0) | 0.988 | 0.128 | 0.150 |
| MCHC  (g/L) | 332.0  (324.8-334.2) | 331.5 (322.0-337.0) | 329.0 (323.8-333.2) | 331.0  (327.8-334.2) | 0.286 | 0.737 | 0.653 |
| Thrombocytes  (x 10^9^/L) | 249.0  (225.8-273.2) | 243.0 (219.8-268.2)* | 255.0 (236.5-272.8) | 250.0  (232.5-270.0)* | **0.006** | 0.464 | 0.965 |
| Leukocytes   (x 10^9^/L) | 5.45  (4.80-6.63) | 6.05  (4.78-6.95) | 5.50  (4.88-6.63) | 6.85  (5.48-8.63)* | **0.001** | **0.004** | **0.003** |
| Neutrophils  (x 10^9^/L) | 2.70  (2.28-3.73) | 3.20  (2.65-4.15)* | 2.95  (2.20-3.43) | 4.35  (3.48-5.93)* | **<0.001** | **<0.001** | **<0.001** |
| Lymphocytes  (x 10^9^/L) | 1.95  (1.68-2.40) | 1.80  (1.58-2.43) | 2.00  (1.68-2.53) | 1.60  (1.40-2.10)* | **<0.001** | 0.104 | **0.006** |
| Monocytes  (x 10^9^/L) | 0.49  (0.42-0.60) | 0.50  (0.41-0.63) | 0.48  (0.39-0.54) | 0.48  (0.41-0.59) | 0.482 | 0.265 | 0.369 |
| Eosinophils  (x 10^9^/L) | 0.11  (0.08-0.17) | 0.13  (0.05-0.19) | 0.09  (0.06-0.14) | 0.08  (0.06-0.13) | 0.188 | 0.157 | 0.163 |
| Basophils  (x 10^9^/L) | 0.040  (0.03-0.06) | 0.045  (0.03-0.05) | 0.050  (0.04-0.06) | 0.040  (0.04-0.06) | 0.477 | **0.045** | 0.290 |

*p<0.05 vs. pre-trial measurement

RBC, red blood cell count; HCT, hematocrit; MCV, mean cell volume; MCH, mean cell hemoglobin; MCHC, mean cell hemoglobin concentration

**Table 2** Comparison of participant perception of thermal conditions in the environmental chamber between exposures, rest vs. exercise in -15 °C. Data presented as median (IQR). Significant p-values in bold.

|  |  | Rest | Exercise | P-value^a^ |
| --- | --- | --- | --- | --- |
| How are you feeling right now?  1=extremely cold, 9=extremely warm | Warm-up^b^ | 5.0  (4.0-5.0) | 4.0  (4.0-5.0) | 0.086 |
|  | Running  interval 1^c^ | 4.0  (3.0-4.0) | 4.0  (4.0-6.0) | **<0.001** |
|  | Running  interval 2^d^ | 3.0  (2.0-4.0) | 5.0  (4.0-6.0) | **<0.001** |
| Are you finding this…?  1=comfortable, 5=extremely uncomfortable | Warm-up^b^ | 1.0  (1.0-2.0) | 2.0  (1.0-2.0) | **0.008** |
|  | Running  interval 1^c^ | 2.0  (2.0-2.0) | 2.0  (1.0-2.0) | **0.005** |
|  | Running  interval 2^d^ | 3.0  (2.0-3.0) | 1.0  (1.0-2.0) | **<0.001** |
| In this moment, are you wishing you were…?  1=much cooler, 7=much warmer | Warm-up^b^ | 5.0  (4.0-5.0) | 5.0  (5.0-5.0) | 0.188 |
|  | Running  interval 1^c^ | 5.0  (5.0-6.0) | 5.0  (4.0-5.0) | **0.007** |
|  | Running  interval 2^d^ | 6.0  (5.0-6.0) | 5.0  (4.0-5.0) | **<0.001** |
| How would you, personally, describe the chamber environment (climate wise)?  1=very acceptable, 4=very unacceptable | Warm-up^b^ | 3.0  (3.0-4.0) | 3.0  (3.0-3.0) | **0.025** |
|  | Running  interval 1^c^ | 3.0  (3.0-3.0) | 3.0  (3.0-3.0) | 0.393 |
|  | Running  interval 2^d^ | 3.0  (2.0-3.0) | 3.0  (3.0-3.0) | **0.019** |
| According to you, is the environment…?  1=completely bearable, 5=completely unbearable | Warm-up^b^ | 1.0  (1.0-1.0) | 1.0  (1.0-2.0) | 0.129 |
|  | Running  interval 1^c^ | 1.0  (1.0-2.0) | 1.0  (1.0-2.0) | 0.091 |
|  | Running  interval 2^d^ | 2.0  (1.0-3.0) | 1.0  (1.0-2.0) | **0.006** |

^a^Comparison rest vs. exercise

^b^After warm-up

^c^After first running interval

^d^After second running interval
